# Dry Needling in Sports and Sport Recovery: A Systematic Review with an Evidence Gap Map

**DOI:** 10.1007/s40279-025-02175-9

**Published:** 2025-02-10

**Authors:** Adrian Kużdżał, Robert Trybulski, Jarosław Muracki, Sebastian Klich, Filipe Manuel Clemente, Adam Kawczyński

**Affiliations:** 1 Institute of Physiotherapy, Faculty of Health Sciences and Psychology, Collegium Medicum, University of Rzeszów, Rejtana Street 16C, 35-959 Rzeszów, Poland; 2 Department of Medical Sciences, The Wojciech Korfanty School of Economics, 40-065 Katowice, Poland; 3 Institute of Physical Culture Sciences, Faculty of Health and Physical Education, University of Szczecin, 70-453 Szczecin, Poland; 4 Department of Sport Didactics, Wrocław University of Health and Sport Sciences, 51-612 Wrocław, Poland; 5 Escola Superior Desporto e Lazer, Instituto Politécnico de Viana do Castelo, Rua Escola Industrial e Comercial de Nun’Álvares, 4900-347 Viana do Castelo, Portugal; 6 Department of Biomechanics and Sport Engineering, Gdańsk University of Physical Education and Sport, 80-336 Gdańsk, Poland

## Abstract

**Background:**

Dry needling is an increasingly popular technique used in sports and regenerative medicine contexts. However, there is no comprehensive overview of investigations of dry needling in sports and sport recovery.

**Objectives:**

The objectives were to perform a systematic review of dry needling in sports athletes with an evidence gap map, to identify current gaps in the literature, and to provide stakeholders with direction for future research.

**Methods:**

Preferred Reporting Items for Systematic Reviews and Meta-analyses (PRISMA) 2020 guidelines were followed. Studies on healthy and injured athletes receiving dry needling were included. Three databases (PubMed, Scopus, and Web of Science) were searched, data were synthesized narratively, key data were summarized quantitatively, and an evidence gap map was created.

**Results:**

The authors incorporated 24 studies into the current study, encompassing 580 athletes, predominantly involving talent/developmental and highly trained/national-level athletes across 13 sports. Most studies used passive or placebo controls, with 37.5% incorporating active controls. Interventions focused mainly on the lower limbs (58.3%). Around 69% of studies reported pain perception outcomes, while six examined muscular strength, activity, and range of motion. While results varied, dry needling generally showed a more positive effect on pain than on athletic performance.

**Conclusions:**

Dry needling studies seem to describe general effectiveness and safety for reducing pain and muscle stiffness in a wide variety of body regions. However, further research is needed on underrepresented populations such as elite, world-class, and Paralympic athletes, as well as expanding investigations into long-term effects and a broader range of muscle groups, particularly the hamstrings. The results may be valuable for medical professionals, sports medicine specialists, and researchers.

**Registration:**

OSF project no.: osf.io osf.io/h3yeq.

**Supplementary Information:**

The online version contains supplementary material available at 10.1007/s40279-025-02175-9.

## Key Points

**Table:** 

Dry needling has emerged as a promising technique within the realm of sports medicine, showing beneficial effects in alleviating pain perception, particularly among athletes in recovery stages.
However, our analysis suggests that dry needling may not offer significant advantages for improving functional performance in athletes.
We identified significant gaps in the existing literature, particularly concerning long-term epidemiological studies and the determination of individualized dosage requirements.
Experimental studies often lack detailed information on dosage and implementation procedures, as well as potential adverse effects, highlighting the need for further research in this area.

## Introduction

Dry needling is a therapeutic technique that involves the insertion of thin, solid needles into myofascial trigger points or tight bands of skeletal muscle [1]. According to the American Physical Therapy Association, dry needling is “a skilled intervention using a fine needle to penetrate the skin and stimulate underlying muscle and connective tissues to manage neuromuscular and movement deficits” [2]. Although it does not follow the exact same techniques as acupuncture, dry needling targets myofascial trigger points in a manner similar to how acupuncture in traditional Chinese medicine treats acupoints, including primary channel points, extra points, and Ah-shi points, to release tension and alleviate pain [3]. The needles are typically the same as those used in acupuncture, but the approach focuses on addressing muscular issues [4]. In sports, dry needling is often employed to enhance athletic performance, treat musculoskeletal injuries, and manage pain [5]. Dry needling targets specific muscle knots or trigger points to improve range of motion, reduce muscle stiffness tone and pain, increase muscle hyperemia [6], improve motor system mobility [7], and promote recovery, making it a valuable adjunct to sports rehabilitation and performance optimization programs.

The increase in training intensity and the congestion of competition schedules have led to a rise in muscle strain, concurrently diminishing the capacity for thorough recovery between sessions and competitions [8, 9]. Excessive mechanical strain on skeletal muscles resulting from the execution of static and/or dynamic physical exercises can lead to myocyte membrane damage and can trigger inflammatory processes, induce pain sensations, and increase muscle stiffness and tension, concurrently reducing muscle elasticity [10–12]. Muscular fatigue induced by training can cause the formation of trigger points that impair muscle function, including increased muscle stiffness and reduced muscle strength [13].

Dry needling is among the potential therapeutic strategies employed to enhance the recovery of athletes, promote their well-being, and potentially aid in injury management [14]. It is considered a safe procedure that does not involve the administration of drugs or other substances [15], eliminating the side effects typically associated with medication use. This makes dry needling an interesting option for athletes recovering from injuries. For instance, Velázquez-Saornil et al. [16] used dry needling in nonathletes as part of rehabilitation following surgical treatment for a complete rupture of the anterior cruciate ligament. Their findings revealed that this treatment approach led to improvements in both the range of motion and the functionality of the operated limb [16]. Kamali et al. [17] also explored the use of the dry needling technique to treat unilateral impingement syndrome of the shoulder joint. This method was effective in reducing pain symptoms, increasing the pain threshold, and improving the mobility of the diseased joint [17]. Moreover, a meta-analysis by Hu et al. [18] showed that the dry needling technique reduces the severity of pain symptoms and increases the volume of movement in athletes with low back pain. Finally, Halle et al. [19] utilized dry needling in post-surgical rehabilitation in nonathletes; this approach demonstrated comparable effectiveness to standard physiotherapy.

In addition to its potential benefits for athletes recovering from injuries, dry needling can also be beneficial for athletes in the recovery process after training. For instance, Walsh et al. [20] noted the positive impact of dry needling on pain intensity after a training period. Sánchez-Infante et al. also confirmed pain reduction after athletes underwent this technique following physical exertion [21]. Moreover, Haser et al. [22] discovered the beneficial effects of this treatment method on range of motion in lower limb joints as well as the strength of the examined muscle group following the procedure.

Despite the growing number of publications dedicated to dry needling in sports performance and injuries, the existing literature lacks effective mapping within a single article. A systematic review presents an effective approach for comprehensively mapping current evidence, summarizing findings, and identifying key gaps in the literature [23]. Combining a systematic review with an evidence gap map can go beyond traditional result pooling and analytical comparisons. This approach enables a visual mapping of evidence, offering a clearer understanding of research trends and highlighting what is known and unknown (i.e., research gaps) about dry needling in athletes [24]. Such valuable insights can inform future policies and funding decisions.

A brief search on PubMed (employing “dry needl*” AND “sport*” OR “exercise*”) yielded 51 records from inception to 2014 and 324 records from 2015 to 2024. This indicates that the majority of studies on dry needling have been published in the last decade, underscoring the rapid growth of research on this topic. Hence, this study’s objectives were to conduct a systematic review and to provide an evidence gap map of dry needling studies in athletes. This approach aimed to identify trends and pinpoint gaps in the existing literature to guide stakeholders in setting priorities for future research.

## Methods

### Registration

This systematic review adhered to the Preferred Reporting Items for Systematic reviews and Meta-Analyses (PRISMA) 2020 guidelines. The a priori protocol registration was conducted on the Open Science Framework (OSF) platform and assigned the project no. osf.io osf.io/h3yeq.

### Equity, Diversity, and Inclusion Statement

This systematic review did not exclude any specific sex or group. As long as individuals were athletes, they were considered for inclusion in the study, regardless of sex, disability status, or any other potential demographic. Being a systematic review, we were unable to ensure a balanced number of studies included. However, we have included all studies that met the eligibility criteria.

### Eligibility Criteria

Our systematic review includes original articles from peer-reviewed journals, even those with “ahead-of-print” status, with a deliberate omission of language restrictions to ensure a comprehensive article selection. Eligibility criteria were established on the basis of the Participants, Intervention, Comparators, Outcomes, and Study Design (PICOS) framework:

- Participants (P): Inclusion criteria covered sports athletes of any age, sex, or sport, competing at a level corresponding to tier two (trained/developmental) or higher of the Participant Classification Framework (PCF). Tiers zero and one, which represent individuals outside of the athlete category, were excluded. Studies involving injured athletes (e.g., rehabilitation or return to sports) or disabled athletes (e.g., cerebral palsy) were also eligible, with no specified minimum number of participants per study.
- Intervention (I): We considered acute (single or multiple sessions with assessments up to 72 h post-intervention) and chronic (multiple sessions with pre- to post-intervention differences assessments) interventions utilizing any form of dry needling strategies. We refrained from predefining a minimum intervention length for chronic categorization, acknowledging that thresholds may vary depending on specific outcomes and intervention characteristics.
- Comparators (C): While not mandatory, comparators were considered, recognizing that we were not directly comparing dry needling effectiveness or efficacy. If available, comparators could include other therapeutic approaches or passive controls.
- Outcomes (O): Inclusion required at least one of the following outcomes or adaptations: acute or chronic physiological, biomechanical, psychological, or performance-related outcomes, and/or data on injury risk or occurrence.
- Study Design (S): All types of experimental and observational studies, including single- or multi-arm, randomized (parallel, crossover, cluster, or other), or nonrandomized studies, were included.

To ensure an appropriate evaluation, a comprehensive review of the full texts was conducted to determine their eligibility for inclusion in this review.

### Information Sources

We employed a comprehensive strategy to identify relevant studies by conducting an extensive search across three key databases: (1) PubMed, (2) Scopus, and (3) Web of Science, up to 11 January 2024. In an effort to enhance the thoroughness of our methodology and minimize the possibility of missing pertinent materials, we also performed manual searches within the reference lists of the studies incorporated into our review.

### Search Strategy

Utilizing the Boolean operators AND/OR, our search strategy deliberately avoided imposing filters or constraints related to publication dates or language. This decision aimed to increase the likelihood of uncovering relevant studies. All included terms were systematically searched within the title and abstract of the selected databases. Notably, in PubMed and Scopus, the keywords were additionally integrated as search options. Meanwhile, within the Web of Science—Core Collection, the terms were specifically chosen to align with topics.

The specific line of code employed for executing these searches is as follows: “dry needl*”[Title/Abstract] AND (“sport*”[Title/Abstract] OR “athletic performance*”[Title/Abstract] OR “exercise*”[Title/Abstract] OR “athletic injur*”[Title/Abstract]).

### Selection Process

Two authors performed the screening process independently, reviewing both titles and abstracts of the retrieved records. Following this initial assessment, each author individually evaluated the full texts of the selected records. In cases of discrepancies during the evaluation, a collaborative reevaluation process was initiated to reach consensus. If consensus could not be reached, the final decision was deferred to a third author.

For efficient record management, we utilized EndNote X9.3.3 software, developed by Clarivate Analytics in Philadelphia, Pennsylvania, USA.

### Data Collection Process

Two authors autonomously conducted the data collection process, ensuring independent assessments. In instances of disagreements during this phase, a third author served as a mediator to resolve any discrepancies. To enhance efficiency and maintain organizational coherence throughout this procedure, a dedicated Microsoft^®^ Excel datasheet was utilized. This datasheet comprehensively incorporated all relevant data and essential information, providing a structured and effective approach to data management.

### Assessment of Risk of Bias

In the context of the randomized experimental studies considered in this current systematic review, we evaluated the risk of bias using the Physiotherapy Evidence Database (PEDro) scale. This scale has been previously validated and confirmed for its reliability [25, 26]. Composed of 11 items, the scale assesses various aspects, including randomization, concealed allocation, baseline comparability, blinding of participants and assessors, and statistical reporting.

Each item is scored as either present or absent, contributing to a total score ranging from 0 to 10, excluding one item (eligibility criteria) which is not scored. Two researchers independently conducted the assessment of the experimental studies included in this systematic review. Following the evaluation, they compared their respective ratings. In the presence of a third author, they collectively determined the final score and risk of bias for each study.

### Data Items

The data collection process included the extraction of a set of participant details and contextual factors, incorporating variables such as publication date, primary research objectives, sample size, country of origin, age distribution, sex, study design specifics, and the competitive level of participants.

Concerning intervention-related conditions, the authors documented information pertaining to the study design and duration, training context, and various aspects of the employed dry needling strategy. Additionally, details from both the active and control groups were extracted, encompassing therapeutic-related information.

The primary emphasis during the extraction of main outcomes centered on parameters associated with acute responses, specifically those related to pain (e.g., soreness), fatigue, and recovery (e.g., perception of recovery, range of motion, muscle architecture). Furthermore, aspects of physical readiness (e.g., athletic performance outcomes) were considered. Chronic responses in athletic performance were also included in our data extraction.

### Data Synthesis Methods

We conducted a narrative synthesis complemented by data summaries, which included numerical representations such as numbers and percentages for the specified data items. To present a comprehensive view of the current state of research and identify areas with limited evidence, we created an evidence gap map. This visual representation was designed to offer an intuitive overview of both the available evidence and the prevailing research gaps.

## Results

### Selection of Sources of Evidence

**Fig. 1:**
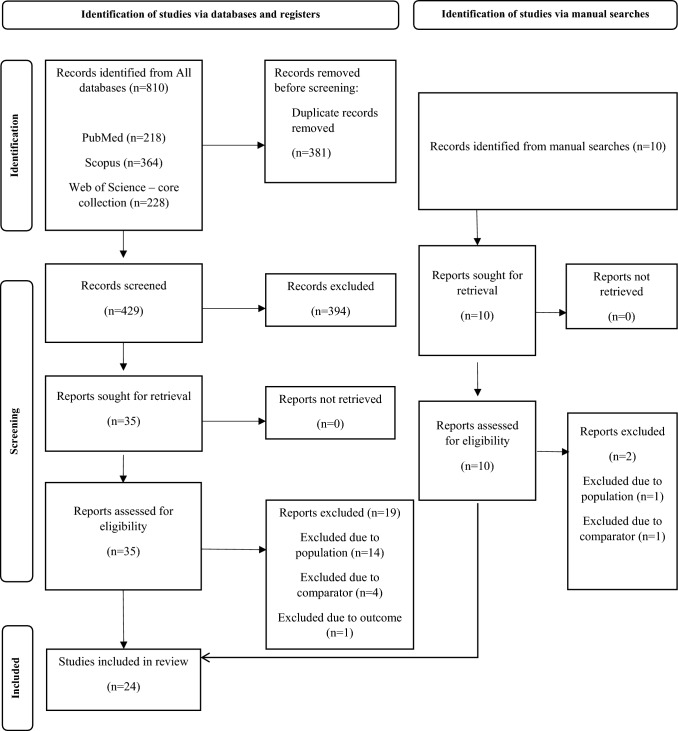
Preferred Reporting Items for Systematic Reviews and Meta-analyses (PRISMA) flow diagram

Figure 1 shows the outcome of the initial database searches, yielding a total of 810 documents. Following the screening process, we identified 16 studies that conformed to our pre-established eligibility criteria. In conjunction with our database screening, we conducted a manual investigation into the references cited in the chosen articles. This supplementary exploration unveiled an additional eight articles that met our inclusion criteria. Consequently, our systematic review includes a total of 24 articles. For a thorough breakdown of the complete full-text screening process and an explanation of exclusions, readers are directed to Supplementary Material 1.

### Assessing the Risk of Bias in Randomized Experimental Studies

Table 1 displays the evaluation of bias risk in the 17 experimental studies included in the present systematic review. Among the examined elements, blinding to participants and therapists emerged as the most crucial aspects, with only 18% of the studies employing this procedure. Similarly, allocation concealment was observed in only 41% of the studies, as indicated in the provided reports. Regarding blinding to evaluators, 59% of the studies reported the incorporation of such a procedure.

**Table 1:** Physiotherapy Evidence Database (PEDro) scale ratings

Study	C1	C2	C3	C4	C5	C6	C7	C8	C9	C10	C11	Score
Aidar et al. [40]	1	1	0	0	0	0	0	1	1	1	1	5
Benito-de-Pedro et al. [27]	1	1	1	1	0	0	1	1	1	1	1	8
Benito-de-Pedro et al. [28]	1	1	1	1	0	0	1	1	1	1	1	8
Benito-de-Pedro et al. [29]	1	1	1	1	0	0	1	1	1	1	1	8
Ceballos-Laita et al. [38]	1	1	1	1	0	0	1	1	1	1	1	8
Cushman et al. [31]	1	1	0	1	1	0	0	1	1	1	0	6
Devereux et al. [33]	1	1	0	1	0	0	0	1	1	1	1	6
Dos Santos et al. [39]	1	1	0	1	0	0	0	1	1	1	1	6
Etminan et al. [45]	1	1	0	1	0	0	0	1	1	1	1	6
Haser et al. [22]	1	1	0	1	0	0	1	1	1	1	1	7
Huguenin et al. [34]	1	1	1	1	1	1	1	1	1	1	1	10
Janowski et al. [35]	1	1	1	1	0	1	1	1	1	1	1	9
Kamali et al. [17]	1	1	0	1	0	0	1	1	1	1	1	7
Kheradmandi et al. [46]	1	1	0	1	0	0	1	1	1	1	1	7
López-González et al. [36]	1	1	0	1	1	0	0	1	1	1	1	7
Walsh et al. [20]	1	1	0	1	0	0	1	1	1	1	1	7
Zarei et al. [7]	1	1	1	1	0	1	0	1	1	1	1	8

*C1* eligibility criteria were specified, *C2* subjects were randomly allocated to groups, *C3* allocation was concealed, *C4* the groups were similar at baseline regarding the most important prognostic indicators, *C5* there was blinding of all subjects, *C6* there was blinding of all therapists who administered the therapy, *C7* there was blinding of all assessors who measured at least one key outcome, *C8* measures of at least one key outcome were obtained from more than 85% of the subjects initially allocated to groups, *C9* all subjects for whom outcome measures were available received the treatment or control condition as allocated, or where this was not the case, data for at least one key outcome were analyzed according to “intention to treat”, *C10* the results of between-group statistical comparisons were reported for at least one key outcome, *C11* the study provided both point measures and measures of variability for at least one key outcome; score, the score was derived by summing the scores from C2 to C11, as recommended by the PEDro scale

### Characteristics of Sources of Evidence

Among the 24 studies included in the analysis (Table 2), the majority (*n* = 15, 62.5%) were conducted with tier two athletes (talent/developmental), five (20.8%) with tier three athletes (highly trained/national level), and one (4.2%) with tier four athletes (elite/international). The remaining three studies (*n* = 3, 12.5%) could not be classified owing to the absence of information. Regarding the sex of participants, 9 (37.5%) studies exclusively focused on men, 3 (12.5%) exclusively focused on women, and the remaining 10 (41.7%) studies included both men and women. Additionally, two studies did not provide a description of the sex distribution. Among the 17 (70.8%) experimental studies included, all were randomized, and seven (29.2%) case reports were also identified. In terms of outcomes, 18 (75%) studies reported measures associated with pain perception, while 14 (58.3%) studies addressed physiological and physical aspects related to the intervention.

**Table 2:** Characteristics of the included studies

Study	Sport	Competitive level	*N*	Sex	Age, years	Randomization	Study design	Parallel group or condition	Length of intervention	Number of sessions	Aim of intervention	Outcomes analyzed
Aidar et al. [40]	Paraplegic powerlifting	Tier 3	12	M	25.4 ± 3.3	Yes	Experimental, crossover	Cold-water immersion, passive recovery	4 weeks	1 per condition	Compare the influence of different recovery methods on postexercise hemodynamic response	Systolic and diastolic blood pressure; heart rate; double product; myocardial oxygen
Benito-de-Pedro et al. [27]	Triathletes	Tier 2	34	ND	34.5 ± 5.5	Yes	Experimental, parallel	Ischemic compression technic	1 day	1	Compare the acute effects of DN and ischemic compression technique in the latent myofascial pain syndrome	Pressure pain threshold; skin temperature
Benito-de-Pedro et al. [28]	Triathletes	Tier 2	34	M and W	34.5 ± 5.5	Yes	Experimental, parallel	Ischemic compression technic	1 day	1	Compare the acute effects of DN and ischemic compression technic in the latent myofascial pain syndrome	Superficial electromyographic activity (mean power frequency at 1 m/sg, 1.5 m/sg, and 2.5 m/sg) in lateral and medial gastrocnemius
Benito-de-Pedro et al. [29]	Triathletes	Tier 2	34	ND	ND	Yes	Experimental, parallel	Ischemic compression technic	1 day	1	Compare the acute effects of DN and ischemic compression technic in the latent myofascial pain syndrome	Ankle dorsiflexion range of motion; distribution of dynamic and static plantar pressures by T-Plate
Brewster et al. [30]	Ice hockey	Tier 3	4	M	20–22	No	Case report	None	1 day	1	Analyze the acute effects on DN on postexercise stress	Acute Recovery Stress Scale
Ceballos-Laita et al. [38]	Handball	Tier 3	30	M	22.4 ± 3.7	Yes	Experimental, parallel	Passive recovery	2 months	1	Analyze the effects of DN active myofascial trigger points in the teres major muscle compared with an untreated control group	Internal and external rotation range of motion and strength; glenohumeral internal rotation deficit; external rotation gain; extensibility; pain intensity
Cushman et al. [31]	Running	Tier 2	61	M and W	41.6 ± 12.3	Yes	Experimental, parallel	Sham DN	1 day	1	Determine the effectiveness of a single session of post-race DN in reducing post-race soreness and the frequency of post-race leg cramps in half-marathon and full-marathon runners	Numeric pain rating; cramps; soreness
Dembowski et al. [32]	Pole-vault	Tier 2	1	M	18	No	Case report	None	3 weeks	9	Analyze the effects of DN on injury treatment process	Active straight leg raise; visual analog scale; hamstring strength; knee active range of motion; single leg triple hop; Illinois Agility Test
Devereux et al. [33]	Field sports	Tier 2	40	M	25.6 ± 5.5	Yes	Experimental, parallel	Passive recovery	6 days	6	Analyze the effects of treating latent myofascial trigger points in the lower limb kinetic chain	Jump height, maximum relative power, maximal force and maximal velocity
Dos Santos et al. [39]	Paraplegic powerlifting	Tier 3	12	M	25.4 ± 3.3	Yes	Experimental, parallel	Cold-water immersion, passive recovery	4 weeks	1 per condition	Compare the influence of different recovery methods on postexercise responses	Maximum isometric force; pain pressure; interleukin (IL), IL-2, and IL-4 and interferon gamma
Escaloni et al. [41]	Wrestling	Tier 2	1	W	15	No	Case report	None	8 weeks	ND	Analyze the effects of orthobiologic preparation, tissue-specific treatment, and regenerative rehabilitation for an athlete grappling with a complex shoulder injury	Numeric pain rating scale
Etminan et al. [45]	Overhead athletes	Tier 2	44	M and W	34.9 ± 6.8	Yes	Experimental, parallel	Physiotherapy treatment only	3 weeks	9	Analyze the impact of integrating dry needling into conventional physiotherapy methods to improve grip strength, functionality, and pain relief	Grip strength; pain relieving
Haser et al. [22]	Soccer	Tier 2	30	ND	18.4	Yes	Experimental, parallel	Placebo laser with water pressure massage, passive recovery	4 weeks	4	Compare the effects of DN plus water pressure against control conditions on muscle force, endurance, and range of motion	Knee extension and flexion maximal force and endurance; hip flexion range of motion
Huguenin et al. [34]	Running	ND	52	M	ND	Yes	Experimental, parallel	Placebo (needles were unwrapped)	1 day	1	Examine the impact of DN treatment on athletes experiencing posterior thigh pain referred from gluteal trigger points, focusing on its effects on the straight leg raise, hip internal rotation, and muscle pain	Straight leg raise range of motion; visual analog rating
Janowski et al. [35]	Ballet	Tier 3	11	M and W	ND	Yes	Experimental, parallel	Sham DN	1 day	1	Evaluate the immediate effects of DN on myofascial trigger points by examining skin surface temperature, pain levels, active and passive range of motion, as well as torque production in the triceps surae	Visual analog pain scale score; dorsiflexion range of motion; torque at angular velocity 60, 90 and 120 degrees
Kamali et al. [17]	Overhead athletes	Tier 2	40	M and W	26.9 ± 6.3	Yes	Experimental, parallel	None	3 days	3	Evaluate and compare the effectiveness of upper trapezius DN versus infraspinatus DN in alleviating pain and reducing disability among individuals experiencing shoulder pain	Visual analog scale, pain pressure threshold, and disability in the arm, shoulder, and hand
Kheradmandi et al. [46]	Overhead athletes	Tier 2	40	M and W	32.0 ± 7.6	Yes	Experimental, parallel	Manual therapy	3 days	3	Assess the impact of DN alone and in combination with manual therapy on pain and functional outcomes in athletes exhibiting scapular dyskinesia	Pain (numeric rating scale); scapular dyskinesia; lateral scapular slide test; pressure pain threshold; disability of arm, shoulder and hand
López-González et al. [36]	Basketball	Tier 2	32	M and W	23.0 ± 5.0	Yes	Experimental, parallel	Placebo	1 day	1	Examine the effects of DN versus placebo DN administered to the peroneus longus and tibialis anterior muscles, focusing on neuromuscular control and static postural stability in players with chronic ankle instability	Pre-activation amplitudes of peroneus longus and tibialis anterior using electromyography; center of pressure displacement and sway variability in anterior–posterior medio-lateral directions
Mason et al. [37]	Ballet	Tier 2	1	W	16	No	Case report	None	2 days	2	Examine the impact of DN on subacute posterior knee pain	Pain (numeric rating scale); global rating of change
Osborne et al. [42]	Volleyball	Tier 4	4	W	25 ± 2	No	Case report	None	1–2 days	1–2	Examine the impact of DN on range of movement, strength, and pain	Pain rating index; visual analog scale; McGill Pain Questionnaire
Patrick et al. [43]	Baseball	Tier 2	1	M	20	No	Case report	None	3 weeks	9	Report the effects of multimodal approach to conservative management of a chronic ulnar collateral ligament	Disability of arm, shoulder and hand; numeric pain rating scale
Walsh et al. [20]	Multiple sports	ND	21	M and W	22.8 ± 2.0	Yes	Experimental, parallel	Radial extracorporeal shockwave therapy, control	1 week	3	Analyze the impact of DN and radial extracorporeal shockwave therapy on the severity of latent myofascial trigger points in the vastus lateralis and vastus medialis	Pressure pain threshold
Westrick et al. [44]	Military athlete	ND	1	M	22	No	Case report	None	4 days	4	Describe the utilization of DN as a tool for both diagnosing and treating focal chest wall pain	Patient-specific functional scale; global rating of change score
Zarei et al. [7]	Athletes	Tier 2	40	W	23.9 ± 5.5	Yes	Experimental, parallel	Exercise therapy	4 weeks	20	Assess the effects of exercise therapy in isolation versus exercise therapy combined DN targeting the gluteus medius and quadratus lumborum on pain and functional outcomes in athletes diagnosed with patellofemoral pain	Kujala score; step-down test; pain pressure threshold; modified star excursion balance test; numerical pain rating score

*M* men, *W* women, *DN* dry needling, *ND* not described

Table 3 presents the body regions where dry needling was implemented in the included studies, as well as the characteristics of the needles and their application. Regarding the body regions, 14 studies focused on lower-limbs, including Benito-de-Pedro et al. [27], Benito-de-Pedro et al. [28], Benito-de-Pedro et al. [29], Brewster et al. [30], Cushman et al. [31], Dembowski et al. [32], Devereux et al. [33], Haser et al. [22], Huguenin et al. [34], Janowski et al. [35], López-González et al. [36], Mason et al. [37], Walsh et al. [20], and Zarei et al. [7]. The remaining 10 studies focused on the upper body.

**Table 3:** Description of methodological approaches to dry needling

Study	Body region	Needle characteristics	Orientation	Guiding tube	Procedural description
Aidar et al. [40]	Pectoralis major; anterior deltoid; brachial triceps	0.25 × 40 mm	Perpendicular	ND	Needle applications were conducted using sterile, stainless steel monofilaments inserted perpendicular to the muscles. The needles were secured in place for a duration of 5 min without any manipulation or stimulation during the execution of the in situ technique
Benito-de-Pedro et al. [27]	Triceps surae	0.3 × 50 mm (Agupunt, Madrid, Spain)	ND	ND	The “fast in, fast out” technique was employed, involving a continuous up-and-down movement of the needle within the skin without complete withdrawal. Subsequently, the DN technique was applied, continuing until the triathlete’s tolerance limit was reached or the maximum number of 8–10 insertions was achieved
Benito-de-Pedro et al. [28]	Latent medial and lateral gastrocnemius	0.3 × 50 mm (Agupunt, Madrid, Spain)	ND	ND	The “fast in, fast out” technique was implemented, involving a continuous upward and downward movement of the needle within the skin without complete withdrawal. Subsequently, the DN technique was administered, continuing until it reached the triathlete’s tolerance limit or reached the maximum number of 8–10 insertions
Benito-de-Pedro et al. [29]	Triceps surae	0.3 × 50 mm (Agupunt, Madrid, Spain)	ND	ND	The “fast in, fast out” technique was implemented, involving a continuous upward and downward movement of the needle within the skin without complete withdrawal. Subsequently, the DN technique was administered, continuing until it reached the triathlete's tolerance limit or reached the maximum number of 8–10 insertions
Brewster et al. [30]	Deep peroneal; tibial; saphenous; common peroneal; iliotibial; sural-I; lateral popliteal; inferior gluteal; superior cluneal; posterior cutaneous of L5	0.3 × 15 mm; 0.30 × 50 mm; 0.3 × 75 mm	Perpendicular; inferomedial orientation; 45° angle	ND	Needles were inserted at both anterior and posterior points for a duration of 15 min in all instances. A comprehensive record documenting positive tenderness to palpation, a positive twitch response, and a positive histamine reaction was meticulously maintained for each needle location during every treatment session for all athletes
Ceballos-Laita et al. [38]	Teres major muscle	0.30 × 50 mm	ND	Yes	The intervention was guided by ultrasound. The “fast in, fast out” technique was employed, with the needle repeatedly inserted until the local twitch responses ceased
Cushman et al. [31]	Lateral soleus muscles; bilateral distal vastus; medialis and distal vastus lateralis muscles	ND	ND	Yes	The needle was passed repeatedly until a local twitch response was elicited. This process was iterated until the local twitch response was extinguished, at which point the needle was withdrawn
Dembowski et al. [32]	Hamstring musculature	0.30 × 60 mm	ND	ND	The number of needles employed per session varied from three to six, with each needle being left in place for a duration of 5 min
Devereux et al. [33]	Rectus femoris; gastrocnemius muscles	ND	ND	ND	The intervention entailed DN in the targeted area, aiming to elicit a local twitch response
Dos Santos et al. [39]	Pectoralis major; anterior deltoid; brachial triceps	0.25 × 40 mm	Perpendicular	ND	Inserted perpendicular to the muscles, the needles were securely held in place for 5 min without any manipulation or stimulation during the execution of the in situ technique
Escaloni et al. [41]	Shoulder	ND	ND	ND	Electrical dry needling was employed
Etminan et al. [45]	Tendon of muscles of the forearm and the fingers	ND	Parallel	ND	The needle was inserted parallel to the skin position, directed toward the radius bone at the origin of the common extensor muscles, and it remained in place for a duration of 15 min
Haser et al. [22]	Thigh muscle	ND	ND	ND	Once the needle triggered a local twitch response, it was promptly removed, concluding the process in 20 min
Huguenin et al. [34]	Gluteal region	0.30 × 25 mm long (Seirin Corp, Shizuoka, Japan)	ND	ND	The indication of recognizable pain or observation of a local twitch response signified appropriate needle placement. Subsequently, the needle was partially withdrawn and iteratively advanced into the muscle until the pain subsided, and no additional twitches were observed. This process was allotted 1 min of treatment per each point
Janowski et al. [35]	Triceps surae	30 × 30 mm (Seirin J-Type)	ND	ND	Needles were inserted into the muscle and moved up and down repeatedly to induce a twitch response
Kamali et al. [17]	Shoulder	0.2 × 50 mm	Perpendicular	Yes	Despite the patient being in a prone position, a skilled physical therapist grasped the taut band between their thumb and index fingers. They then systematically needled forward and backward into the trigger point until no more local twitch responses occurred
Kheradmandi et al. [46]	Subscapularis; pectoralis minor; serratus anterior; upper and lower trapezius muscles	ND	ND	ND	ND
López-González et al. [36]	Peroneus longus; tibialis anterior	0.25 × 50 mm (APS Agu-Punt)	Perpendicular	ND	The Hong technique was applied at a frequency of 1 Hz for 30 s (1 puncture per second). Following the initial twitch response, the needle was vertically displaced by 2–3 mm at the same frequency
Mason et al. [37]	Gastrocnemius muscle; popliteus muscle	0.25 × 50 mm	ND	ND	Upon insertion into the skin, the needle was directed toward the target MTP and systematically “pistoned”: inserted and withdrawn from each MTP without being fully withdrawn from the skin. This process aimed to elicit local twitch responses
Osborne et al. [42]	Shoulder	0.25 × 40 mm (Dongbang Acupuncture needles)	Perpendicular	ND	Between 5 and 12 needles were inserted into the muscles, oriented perpendicular to the fibers, with a deep insertion ranging from half to two-thirds of the entire length of the needle’s shaft. Each needle was rotated until local tenderness was perceived, and a referred sensation to the anterior aspect of the shoulder was noted, after which they were left in place for 10 min
Patrick et al. [43]	Flexor carpi radialis; flexor digitorum superficialis; flexor digitorum profundus	ND	ND	ND	The needles were left in the target tissues and connected to electrical stimulation (2 Hz, using the ITO ES-130 Three-Channel Electro Stimulation Unit from Superior Medical Equipment in Wilmington, NC, USA). This was done to induce a localized twitch response, and the needles were left in position for a duration of 8 min
Walsh et al. [20]	Vastus lateralis; vastus medialis	0.30 × 60 mm (L-typeTM; Seirin Corporation, Shizuoka, Japan)	ND	ND	The sensitive loci within the myofascial trigger points were dynamically stimulated using the “fast-in, fast-out technique” for 30 s. The trigger point stimulation was then continued in 30-s increments until the local twitch response was no longer present or for a maximum duration of 2 min
Westrick et al. [44]	Chest	ND	ND	ND	Dry needling was conducted over the second rib at the costochondral joint and along the superficial soft tissues corresponding to the second rib
Zarei et al. [7]	Quadratus lumbarum; gluteus medius	0.25 × 50 mm; 0.30 × 100 mm	ND	ND	The “fast-in, fast-out” technique was administered with the patient in a side-lying position, repeated three times for each myofascial trigger point

DN, dry needling; ND, not disclosed

### Results of Individual Sources of Evidence

Table 4 presents the main results extracted from studies reporting participants’ pain intensities before and after exposure to dry needling.

**Table 4:** Summary of pain results for the included studies

Study	Outcome	Pre-DN	Post-DN	Percentage of difference (post- and pre-DN)	Main findings
Benito-de-Pedro et al. [27]	Pressure pain threshold (A.U.)	2.63 ± 0.65	1.94 ± 0.58	− 26.2	Following the treatment, statistically significant differences (*p* > 0.05) were observed, indicating a reduced pressure pain threshold in athletes who underwent DN compared with those who received ischemic compression immediately after the treatment
Brewster et al. [30]	ARSS: physical performance capability (A.U.)	5.6 ± 0.4	5.2 ± 0.9^a^	− 7.1	At the 24-h and 48-h post-assessment points, four subcategories of the ARSS demonstrated the most consistent positive changes, particularly noteworthy, as these assessments coincided with periods of elevated training loads in collegiate ice hockey players. Conversely, two subcategories within the stress scale exhibited limited consistency across cases at the 24-h mark but displayed more consistent positive improvements by the 48-h post-assessment. Notably, two subcategories within the recovery scale demonstrated minimal changes and consistency among athletes at both the 24-h and 48-h post-assessment intervals
Brewster et al. [30]	ARSS: emotional balance (A.U.)	5.4 ± 0.4	5.4 ± 0.9^a^	0.0	
Brewster et al. [30]	ARSS: mental performance capability (A.U.)	5.5 ± 0.5	5.4 ± 0.9^a^	− 1.8	
Brewster et al. [30]	ARSS: overall recovery (A.U.)	4.9 ± 0.7	4.9 ± 0.9^a^	0.0	
Brewster et al. [30]	ARSS: muscular stress (A.U.)	1.1 ± 0.5	1.4 ± 0.5^a^	27.3	
Brewster et al. [30]	ARSS: negative emotional state (A.U.)	0.5 ± 0.4	0.8 ± 1.0^a^	60.0	
Brewster et al. [30]	ARSS: lack of activation (A.U.)	1.0 ± 0.4	0.6 ± 0.8^a^	− 40.0	
Brewster et al. [30]	ARSS: overall stress (A.U.)	0.9 ± 0.8	1.4 ± 0.9^a^	55.6	
Ceballos-Laita et al. [38]	Pain intensity (A.U.)	4.0 ± 2.2	0.7 ± 0.7	− 82.5	The group undergoing DN exhibited greater changes in pain intensity compared with the control group
Cushman et al. [31]	Pain score (A.U.): left soleus	3.5 ± ND	0.3 ± ND^b^	− 91.4	This study did not reveal any enhancement in delayed-onset muscle soreness or delayed-onset muscle cramping among half-marathon and full-marathon runners following a single session of immediate postrace dry needling when compared with sham DN
Cushman et al. [31]	Pain score (A.U.): right soleus	3.3 ± ND	0.3 ± ND^b^	− 90.9	
Cushman et al. [31]	Pain score (A.U.): left quadriceps	3.3 ± ND	0.5 ± ND^b^	− 84.9	
Cushman et al. [31]	Pain score (A.U.): right quadriceps	4.4 ± ND	0.5 ± ND^b^	− 88.6	
Cushman et al. [31]	Pain score (A.U.): left hamstrings	3.2 ± ND	0.3 ± ND^b^	− 90.6	
Cushman et al. [31]	Pain score (A.U.): right hamstrings	4.0 ± ND	0.5 ± ND^b^	− 87.5	
Dembowski et al. [32]	Visual analog scale (A.U.)	32.0 ± 25.0^a^	24.7 ± 15.0^c^	− 22.8	Following the intervention sessions, there was a slight reduction in pain
Dos Santos et al. [39]	Pain pressure: pectoralis sternal part (A.U.)	6.2 ± 1.4	5.4 ± 1.2^a^	− 12.9	In the DN recovery method, a comparable trend to passive recovery was observed, albeit with a general decrease in pain pressure threshold that was less pronounced than that seen in passive recovery
Dos Santos et al. [39]	Pain pressure: triceps (A.U.)	6.0 ± 0.9	4.7 ± 1.1^a^	− 21.7	
Dos Santos et al. [39]	Pain pressure: pectoralis clavicular part (A.U.)	6.3 ± 1.1	5.6 ± 1.0^a^	− 11.0	
Dos Santos et al. [39]	Pain pressure: anterior deltoid (A.U.)	6.7 ± 0.8	6.6 ± 1.4^a^	− 1.5	
Escaloni et al. [41]	Numeric pain rating scale (A.U.): pain at rest	5.0 ± 0.0	0.0 ± 0.0^e^	− 100.0	Treatment was administered to alleviate pain at rest
Etminan et al. [45]	Pain rate (A.U.)	34.62 ± 9.39	4.00 ± 5.28^f^	− 88.4	DN demonstrated significantly greater effectiveness compared with undergoing physiotherapy alone during the intervention period
Huguenin et al. [34]	Visual analogue rating (A.U.): hamstring tightness	36.6 ± ND	17.7 ± ND^g^	− 51.6	There was no statistically significant difference in the magnitude of change between the groups assigned to either DN or placebo (*p* = 0.013). In the global rating of change analysis, none of the subjects reported feeling worse after treatment. Among the participants, 14 in the placebo group and 18 in the therapeutic group perceived improvement, with 1 subject in the therapeutic group reporting feeling much improved. However, these observations did not result in a statistically significant difference upon analysis
Huguenin et al. [34]	Visual analogue rating (A.U.): hamstring pain	30.6 ± ND	11.8 ± ND^g^	− 61.4	
Huguenin et al. [34]	Visual analogue rating (A.U.): gluteal tightness	28.5 ± ND	9.7 ± ND^g^	− 66.0	
Huguenin et al. [34]	Visual analogue rating (A.U.): gluteal pain	21.5 ± ND	7.0 ± ND^g^	− 67.4	
Janowski et al. [35]	Visual analogue score (A.U.): right side	4.0 ± 1.1	3.4 ± 1.7	− 15.0	There were no statistically significant differences in the mean visual analog scale reported pain scores between either the sham or DN groups from pre- to post-intervention
Janowski et al. [35]	Visual analogue score (A.U.): left side	4.3 ± 1.1	3.7 ± 2.0	− 14.0	
Kamali et al. [17]	Visual analogue scale (A.U.): upper trapezius	6.4 ± 1.8	1.7 ± 1.3	− 73.4	No significant intergroup difference was observed in terms of the visual analog scale (*p* = 0.77). Nevertheless, the results indicated that remote indirect DN proved to be equally effective as direct DN in reducing shoulder pain
Kamali et al. [17]	Visual analogue scale (A.U.): infraspinatus	6.4 ± 1.9	1.5 ± 1.3	− 76.3	
Kheradmandi et al. [46]	Pressure pain threshold (A.U.)	28.19 ± 11.71	27.99 ± 13.42	− 0.7	The combination of DN with manual therapy resulted in a significant reduction in pain compared with manual therapy alone
Kheradmandi et al. [46]	Numeric Rating Scale (A.U.)	8 ± 1.8	3 ± 1	− 62.5	
Mason et al. [37]	Numerical pain score (A.U.)	0–6 ± ND	0 ± ND^b^	ND	The subject reported the complete resolution of symptoms
Osborne et al. [42]	Pain rating index (A.U.)	8.5 ± 7.0	1.8 ± 1.7^b^	− 78.8	These results suggest a trend of decreasing functional pain over the days following treatment
Osborne et al. [42]	Visual analogue scale (A.U.)	4.0 ± 2.0	1.5 ± 1.0^b^	− 62.5	
Osborne et al. [42]	Present pain intensity (A.U.)	2.5 ± 0.6	0.8 ± 0.5^b^	− 68.0	
Patrick et al. [43]	Numerical pain rating scale (A.U.)	10 ± ND	6 ± ND	− 40.0	The pain decreased following the DN intervention
Walsh et al. [20]	Pressure pain threshold (A.U): vastus lateralis (standing leg)	28.9 ± 0.9	25.8 ± 1.0^ h^	− 10.7	DN seems to positively impact the pressure pain threshold of myofascial trigger points in the vastus lateralis and vastus medialis, particularly after the subsidence of post-needling soreness. Radial extracorporeal shockwave therapy demonstrates comparable effectiveness to DN after three treatments, but the improvement between session three and follow-up is less dramatic, possibly attributable to the absence of post-treatment soreness
Walsh et al. [20]	Pressure pain threshold (A.U): vastus lateralis (non-standing leg)	25.7 ± 0.9	26.0 ± 0.9^ h^	1.2	
Walsh et al. [20]	Pressure pain threshold (A.U): vastus medialis (standing leg)	30.1 ± 0.9	28.8 ± 0.9^ h^	− 4.3	
Walsh et al. [20]	Pressure pain threshold (A.U): vastus medialis (non-standing leg)	26.9 ± 1.0	24.3 ± 1.0^ h^	− 9.7	
Westrick et al. [44]	Patient specific functional scale (A.U.): pushups	3	9^d^	200.0	Functionality and pain showed improvement following the DN intervention
Westrick et al. [44]	Patient specific functional scale (A.U.): bench press	5	9^d^	80.0	
Westrick et al. [44]	Patient specific functional scale (A.U.): lifting/twisting	6	10^d^	66.7	
Westrick et al. [44]	Patient specific functional scale (A.U.): equipment run	5	1^d^	− 80.0	
Zarei et al. [7]	Quadratus lumborum pain pressure threshold (A.U.)	3.7 ± ND	7.1 ± ND^h^	91.9	Pain showed significant improvement after the intervention compared with the control group, both immediately after treatment and at the 6-week follow-up
Zarei et al. [7]	Gluteus medius pain pressure threshold (A.U.)	4.3 ± ND	7.7 ± ND^h^	79.1	
Zarei et al. [7]	Numerical pain rating score (A.U.)	5.8 ± ND	1.4 ± ND^h^	− 75.9	

*ARSS* active recovery stress scale, *DN* dry needling, *ND* not described, *A.U.* arbitrary units

^a^Average of the individuals reported after 48 of the intervention

^b^Average reported in the seventh day after intervention

^c^Average of the three sessions reported

^d^Reports after 5 weeks’ intervention

^e^Pain reported after 13 weeks

^f^Pain reported 1 week after the ninth session

^g^Median of pain after 72 h

^h^Report after third intervention

Table 5 presents the main results extracted from studies reporting the physiological and physical responses of participants after exposure to dry needling.

**Table 5:** Summary of range of motion, muscular strength, and physiological results for the included studies

Study	Outcome	Pre-DN	Post-DN	Percentage of difference (post- and pre-DN)	Main findings
Aidar et al. [40]	Systolic blood pressure (mmHg)	122.4 ± 2.8	129.6 ± 8.0^a^	5.9	Dry needling resulted in a decrease in systolic blood pressure immediately after the treatment. However, the opposite trend was noted at 50- and 60-min post-recovery. Significantly higher heart rate and double product values were observed after the passive recovery method, as compared with both cold water and dry needling
Aidar et al. [40]	Diastolic blood pressure (mmHg)	72.1 ± 8.8	72.5 ± 11.6	0.6	
Aidar et al. [40]	Heart rate (bpm)	74.3 ± 13.1	89.4 ± 6.2	20.4	
Aidar et al. [40]	Double product	8980.6 ± 1941.7	11,407.8 ± 12,621.4	27.1	
Aidar et al. [40]	Myocardial oxygen	6.6 ± 2.3	10.0 ± 1.4	51.5	
Benito-de-Pedro et al. [27]	Temperature in superficial area of myofascial trigger point (°C)	31.73 ± 1.99	31.71 ± 1.98	− 0.1	There were no statistically significant differences (*p* > 0.05) in thermography measurements between the two treatment groups, DN, and ischemic compression. This lack of significance was observed both in the superficial zone adjacent to the latent myofascial trigger point and in the corresponding anatomical location with healthy soft tissue in the contralateral limb before and after treatment
Benito-de-Pedro et al. [28]	Electromyography mean power frequency speed of 1 m/sg (%)	33.75 ± 14.84	− 4.46 ± 11.53	− 1113.3	Statistically significant differences (*p* = 0.037) were observed in the reduction of superficial electromyography measurements (%) between the DN and the comparison group at a speed of 1 m/s immediately after both interventions. However, such differences were not evident at speeds of 1.5 m/s or 2.5 m/s
Benito-de-Pedro et al. [28]	Electromyography mean power frequency speed of 1.5 m/sg (%)	34.84 ± 14.43	− 1.00 ± 9.63	− 102.9	
Benito-de-Pedro et al. [28]	Electromyography mean power frequency speed of 2.5 m/sg (%)	38.69 ± 15.19	2.13 ± 7.42	− 94.5	
Benito-de-Pedro et al. [29]	Ankle dorsiflexion with knee extension (°)	16.2 ± 6.1	21.1 ± 6.5	30.2	DN and ischemic compression, when applied to latent myofascial trigger points in the shortened gastrocnemius muscle of triathletes, demonstrated no significant disparities in dorsiflexion range of motion at the tibiofibular–talar joint or in static and dynamic plantar pressure alterations before and immediately after the intervention. The comparable efficacy observed in both treatments suggests that they yield similar results
Benito-de-Pedro et al. [29]	Ankle dorsiflexion with knee flexion (°)	17.7 ± 7.8	19.7 ± 8.5	11.3	
Benito-de-Pedro et al. [29]	Dynamic plantar pressure: surface (cm^2^)	139.2 ± 24.6	140.1 ± 23.2	0.6	
Benito-de-Pedro et al. [29]	Dynamic plantar pressure: maximal pressure (g/cm^2^)	2036.2 ± 237.2	2026.9 ± 322.6	− 0.5	
Benito-de-Pedro et al. [29]	Dynamic plantar pressure: mean pressure (g/cm^2^)	1032.1 ± 144.0	1009.0 ± 141.7	− 2.2	
Benito-de-Pedro et al. [29]	Static plantar pressure: surface (cm^2^)	122.9 ± 19.6	123.9 ± 20.8	0.8	
Benito-de-Pedro et al. [29]	Static plantar pressure: maximal pressure (g/cm^2^)	655.4 ± 119.9	669.8 ± 125.2	2.2	
Benito-de-Pedro et al. [29]	Static plantar pressure: mean pressure (g/cm^2^)	279.3 ± 38.6	288.4 ± 47.3	3.3	
Ceballos-Laita et al. [38]	Internal rotation range-of-motion (°)	22.0 ± 6.5	45.3 ± 6.5	106.8	The group that underwent DN exhibited more significant changes than the control group in internal rotation range of motion (ROM), external rotation ROM, glenohumeral internal rotation deficit, external rotation gain, and extensibility. However, there were no differences between groups or within groups for maximum isometric strength
Ceballos-Laita et al. [38]	External rotation range-of-motion (°)	95.6 ± 10.5	89.1 ± 11.1	− 6.9	
Ceballos-Laita et al. [38]	Glenohumeral internal rotation deficit (°)	− 25.6 ± 8.9	− 2.5 ± 9.0	90.2	
Ceballos-Laita et al. [38]	External Rotation Gain (°)	8.4 ± 7.7	1.9 ± 13.1	− 77.4	
Ceballos-Laita et al. [38]	Internal rotation strength (kg)	13.1 ± 2.0	13.5 ± 2.7	3.1	
Ceballos-Laita et al. [38]	External rotation strength (kg)	16.5 ± 4.0	17.3 ± 4.4	4.8	
Ceballos-Laita et al. [38]	Extensibility (°)	− 14.4 ± 6.7	− 0.8 ± 3.8	94.4	
Dembowski et al. [32]	Active straight leg raise (°)	57.3 ± 4.2^@^	61.3 ± 7.4^b^	7.0	After the three sessions, there were observable improvements on average
Devereux et al. [33]	Jump height (cm): rectus femoris DN	30.8 ± 8.0	31.6 ± 7.4^c^	2.6	The study results indicated a statistically significant increase in jump height specifically within the gastrocnemius DN group, from immediately post-intervention to 48 h post-intervention (*p* < 0.05). No other statistically significant differences were observed
Devereux et al. [33]	Jump height (cm): gastrocnemius DN	30.5 ± 2.5	30.7 ± 2.7	0.7	
Devereux et al. [33]	Jump height (cm): rectus femoris and gastrocnemius DN	30.9 ± 4.8	31.3 ± 4.5	1.3	
Devereux et al. [33]	Maximum power (W/kg): rectus femoris DN	31.9 ± 7.8	31.4 ± 10.3	− 1.6	
Devereux et al. [33]	Maximum power (W/kg): gastrocnemius DN	30.5 ± 2.5	30.7 ± 2.7	0.7	
Devereux et al. [33]	Maximum power (W/kg): rectus femoris and gastrocnemius DN	30.9 ± 4.8	31.3 ± 4.5	1.3	
Devereux et al. [33]	Maximal force (N): rectus femoris DN	28.1 ± 6.2	30.1 ± 6.0	7.1	
Devereux et al. [33]	Maximal force (N): gastrocnemius DN	33.3 ± 5.2	32.4 ± 4.1	− 2.7	
Devereux et al. [33]	Maximal force (N): rectus femoris and gastrocnemius DN	30.9 ± 3.7	33.6 ± 4.7	8.7	
Devereux et al. [33]	Maximal velocity (m/s): rectus femoris DN	4.7 ± 1.7	4.3 ± 1.4	− 8.5	
Devereux et al. [33]	Maximal velocity (m/s): gastrocnemius DN	3.4 ± 0.9	3.5 ± 1.0	2.9	
Devereux et al. [33]	Maximal velocity (m/s): rectus femoris and gastrocnemius DN	3.6 ± 1.3	3.2 ± 1.2	− 11.1	
Dos Santos et al. [39]	Maximal force (N)	851.3 ± 76.5	767.0 ± 49.3^d^	− 10.0	DN was the sole intervention that led to increased IL-2 levels at various time points. Following DN, there was no increase in muscle thickness; conversely, after cold water application, muscle thickness was higher at 15 min and 2 h. The findings suggest that cold water offers effective recovery up to 24 and 48 h later, while dry needling seems to be a favorable option within the first 24 h after the training session
Dos Santos et al. [39]	Cytokines IL-2 (pg/mL)	1.5 ± 0.1	1.9 ± 0.1^d^	26.7	
Dos Santos et al. [39]	Cytokines IL-4 (pg/mL)	1.6 ± 0.1	1.8 ± 0.1^d^	12.5	
Dos Santos et al. [39]	Cytokines IFN^y^ (pg/mL)	1.1 ± 0.1	1.2 ± 0.1^d^	9.1	
Etminan et al. [45]	Grip strength (kg)	1.77 ± 8.79	2.31 ± 7.52^e^	30.5	Concerning the grip strength variable, both groups exhibited a clinically noticeable increase, with a more pronounced enhancement rate observed in the group that received physiotherapy alongside DN. However, no statistically significant difference in the grip strength variable was observed between the two groups at all time intervals (*p* = 0.09)
Haser et al. [22]	Maximal force: knee extension (Nm)	230.7 ± 12.6	226.5 ± 15.3	− 1.8	DN demonstrated noteworthy enhancements in the muscular endurance of knee extensors and hip flexors, persisting for up to 4 weeks post-treatment. Additionally, a short-term improvement in the muscular endurance of knee flexors was observed in the intragroup analysis. Furthermore, when compared with the placebo, DN exhibited significant superiority in enhancing hip flexion
Haser et al. [22]	Endurance: knee extension (Nms)	294.6 ± 15.4	311.0 ± 25.4	5.6	
Haser et al. [22]	Maximal force: knee flexion (Nm)	136.0 ± 11.7	142.7 ± 11.5	4.9	
Haser et al. [22]	Endurance: knee flexion (Nms)	163.5 ± 10.9	188.5 ± 16.3	15.3	
Haser et al. [22]	Hip flexion (°)	81.5 ± 3.3	89.8 ± 2.8	10.7	
Huguenin et al. [34]	Hip internal rotation (°)	31 ± 9	31 ± 8^f^	− 3.2	Neither DN nor placebo needling of the gluteal muscles elicited any alterations in straight leg raise or hip internal rotation
Huguenin et al. [34]	Straight leg raise (°) up to first onset of sensation (stretch or pain)	53 ± 13	55 ± 11	3.8	
Huguenin et al. [34]	Straight leg raise (°) maximum range of motion	72 ± 14	73 ± 15	1.4	
Janowski et al. [35]	Mean temperature (°) of three standard points: right side	96.9 ± 0.2	96.5 ± 0.3	− 0.4	Following the intervention, there was a significant difference in mean temperature for both the right and left calves in the sham group, and the right calf in the DN group. In terms of seated range of motion, the sham group exhibited a statistically larger change in range of motion pre/post-intervention on the left side (*p* = 0.005) compared with the DN group. However, there was no statistical difference in lunge range of motion measurements between the DN and sham groups
Janowski et al. [35]	Mean temperature (°) of three standard points: left side	96.7 ± 0.5	96.4 ± 0.4	− 0.3	
Janowski et al. [35]	Open kinetic chain dorsiflexion range of motion (°): right side	11.3 ± 5.1	12.1 ± 1.5	7.1	
Janowski et al. [35]	Open kinetic chain dorsiflexion range of motion (°): left side	11.1 ± 3.5	10.3 ± 3.6	− 7.2	
Janowski et al. [35]	Closed kinetic chain dorsiflexion range of motion (°): right side	28.2 ± 5.3	29.9 ± 4.0	6.0	
Janowski et al. [35]	Closed kinetic chain dorsiflexion range of motion (°): left side	29.1 ± 5.0	28.3 ± 3.7	− 2.8	
Janowski et al. [35]	Mean triceps surae torque (Nm) at 60°/s: right side	38.9 ± 8.5	45.1 ± 10.8	16.1	
Janowski et al. [35]	Mean triceps surae torque (Nm) at 60°/s: left side	35.6 ± 6.3	43.2 ± 9.0	21.3	
Janowski et al. [35]	Mean triceps surae torque (Nm) at 90°/s: right side	36.3 ± 8.7	37.4 ± 7.9	3.0	
Janowski et al. [35]	Mean triceps surae torque (Nm) at 90°/s: left side	38.9 ± 8.8	36.8 ± 8.9	− 5.4	
Janowski et al. [35]	Mean triceps surae torque (Nm) at 120°/s: right side	34.6 ± 4.8	34.2 ± 8.4	− 1.2	
Janowski et al. [35]	Mean triceps surae torque (Nm) at 120°/s: left side	35.7 ± 9.6	34.2 ± 5.6	− 4.2	
López-González et al. [36]	Pre-activation tibialis anterior (mV)	0.01 ± 0.01	0.09 ± 0.01^ g^	800	Following a single session of DN targeting latent myofascial trigger points (MTrPs) within the tibialis anterior (TA) and peroneus longus (PL) muscles in basketball players with chronic ankle instability (CAI), a significant increase in muscle preactivation was observed for both PL and TA in favor of the DN group during a landing task (*p* < 0.001). Furthermore, those who received DN demonstrated statistically significant improvements in static postural control measures, evidenced by decreased sway variability and center of pressure (CoP) displacement (*p* < 0.001), unlike those who did not undergo DN. These findings, sustained up to 1 month after the intervention, suggest modifications in both feedback and feed-forward strategies following DN of latent MTrPs in PL and TA muscles
López-González et al. [36]	Pre-activation peroneus longus (mV)	0.01 ± 0.01	0.06 ± 0.02^ g^	500	
López-González et al. [36]	Medial–lateral range (mm)	27.8 ± 3.9	14.7 ± 3.3^ g^	− 47.1	
López-González et al. [36]	Anterior–posterior range (mm)	25.4 ± 3.2	16.1 ± 2.3^ g^	− 36.6	
Zarei et al. [7]	Modified star excursion balance test (anterior) mean score	0.75 ± ND	0.9 ± ND	20.0	Only the DN group exhibited clinically significant improvements in functional performance across all three directions. When comparing the two groups, it was evident that the combined approach of exercise therapy and dry needling (DN) resulted in significant additional benefits across all measured outcomes
Zarei et al. [7]	Modified star excursion balance test (posterolateral) mean score	0.74 ± ND	0.9 ± ND	21.6	
Zarei et al. [7]	Modified star excursion balance test (posteromedial) mean score	0.66 ± ND	0.77 ± ND	16.7	

*DN* dry needling

^a^Immediately after intervention

^b^Average of the three sessions reported

^c^Results after 96 h of the intervention

^d^Average of the individuals reported after 48 h of the intervention

^e^Reported 1 week after the ninth session

^f^72 h after intervention

^g^Results of 1 month after intervention

### Evidence Gap Map

**Fig. 2:**
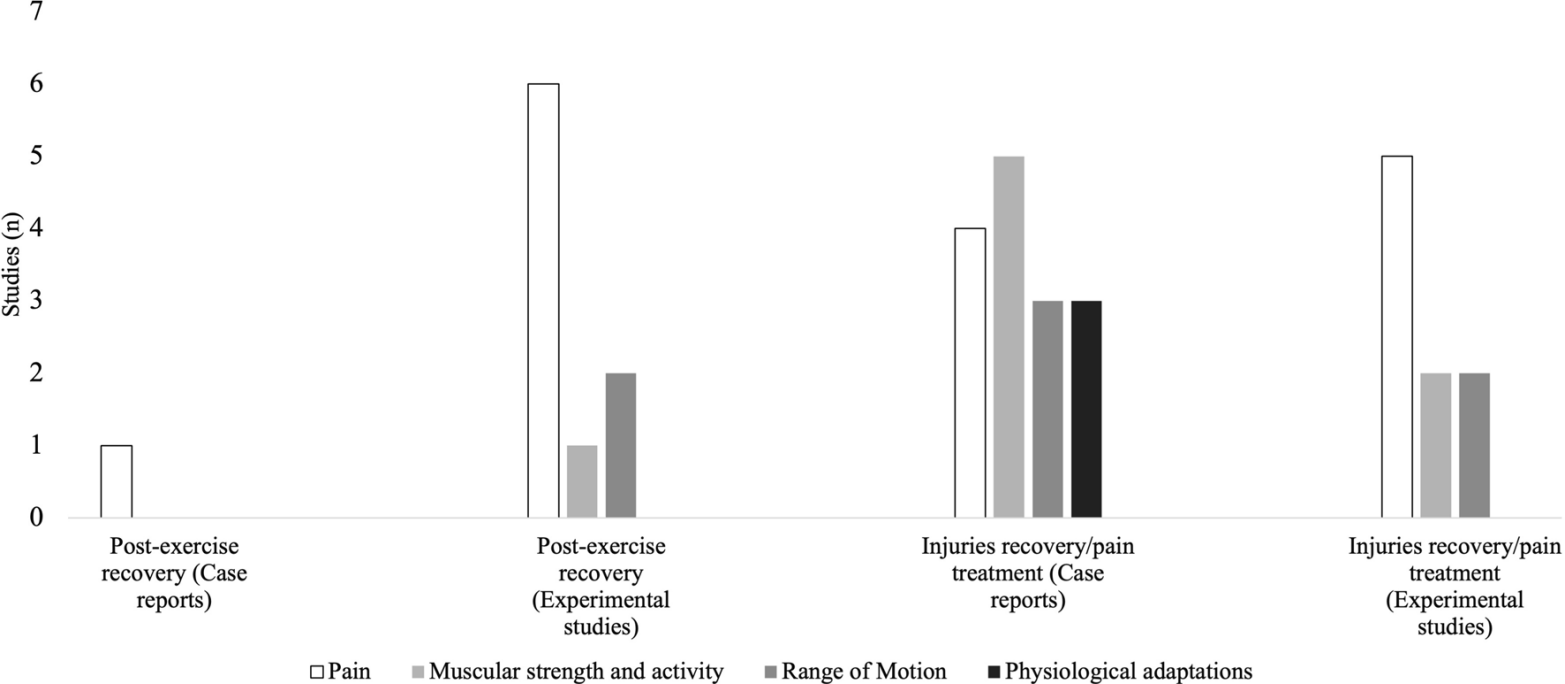
Visualization of the number of studies per topic and study design related to the application of dry needling in athletes. Muscular strength and activity refer to both muscular strength and muscular power, as measured through physical performance tests

Figure 2 illustrates the distribution of studies based on their topics and study designs concerning the application of dry needling in athletes. Specifically focusing on studies related to postexercise recovery, Brewster et al. [30] reported a case focusing on pain, while experimental studies on this topic were conducted by Benito-de-Pedro et al. [27], Cushman et al. [31], Janowski et al. [35], and Walsh et al. [20]. Analyzing muscular strength and activity in postexercise recovery, five experimental studies, namely Benito-de-Pedro et al. [28], Ceballos-Laita et al. [38], Devereux et al. [33], Dos Santos et al. [39], and Haser et al. [22], were identified. Regarding range of motion, four studies reported outcomes Benito-de-Pedro et al. [29], Ceballos-Laita et al. [38], Haser et al. [22], and Janowski et al. [35], while three others presented outcomes related to physiological adaptations, namely Aidar et al. [40], Benito-de-Pedro et al. [27], and Dos Santos et al. [39].

In studies focusing on injury recovery and pain management, six case reports provided outcomes regarding pain: Dembowski et al. [32], Escaloni et al. [41], Osborne et al. [42], Mason et al. [37], Patrick et al. [43], and Westrick et al. [44]. Additionally, five experimental studies also reported pain-related outcomes: Etminan et al. [45], Huguenin et al. [34], Kamali et al. [17], Kheradmandi et al. [46], and Zarei et al. [7]. Concerning muscular strength and activity, one case report (Dembowski et al. [32]) and two experimental studies (Etminan et al. [45] and López-González et al. [36]) presented outcomes. Finally, regarding range of motion, one case report (Dembowski et al. [32]) and two experimental studies, namely Huguenin et al. [34] and Zarei et al. [7], reported outcomes.

## Discussion

Dry needling is creating meaningful developments in sports medicine and physiotherapy practices, expanding its scope beyond traditional clinical settings to enhance athlete recovery and pain management. This systematic review identifies various methodological approaches to dry needling in sports performance and recovery. A multitude of studies investigating these approaches has been observed, with case reports and randomized experimental studies being the most prevalent.

Pain perception emerged as one of the most extensively researched outcomes, regardless of whether the focus was on postexercise recovery or injury rehabilitation. Regarding athletic performance, a wide range of outcomes has been observed, encompassing improvements in range of motion, muscular strength, and physiological responses. Concerning different applications of dry needling, there is a notable emphasis on targeting the lower limbs or shoulders, with relatively few studies concentrating on other regions of the body. The findings exhibit diversity, indicating that, while dry needling shows an important effect on reducing pain, its impact on enhancing athletic performance parameters appears to be less pronounced.

### Methodological Characteristics and Coverage of Dry Needling Studies in Sports

The discussion on the methodological characteristics of dry needling in sports focuses on the populations studied (e.g., sports represented, competitive level, and sex), as well as the specific study designs and outcomes and the context.

#### Populations Included in Dry Needling Studies in Sports

This systematic review has revealed that the majority of studies involving sports athletes focus on talent development and highly trained national-level athletes, highlighting a lack of research on highly competitive athletes at the elite or international/world-class level [47]. Furthermore, Paralympic athletes were only included in two studies, namely those conducted by Aidar et al. [40] and Dos Santos et al. [39]. The larger sample size and less congested competition schedules faced by athletes at smaller competitive levels probably make it easier to conduct experiments. However, the underrepresentation of specific competitive levels (such as elite or world-class) or Paralympic athletes may make it difficult to generalize evidence and ensure the suitability of interventions for these populations, who are subject to high levels of strain and require more comprehensive recovery strategies [48].

Regarding sex representation, most studies included both men and women. Interestingly, among those focusing solely on one sex, there was a notable balance between studies concentrating on men and women. This reflects the importance of achieving a more equitable representation of women as research subjects in sports. One possible explanation for this balance could be the greater equilibrium in the sex of the authors of studies, which appears to influence the ultimate research focus [49]. However, in different contexts, women remain underrepresented, resulting in imbalances in the understanding of interventions concerning the specificities of biological characteristics [50].

#### Study Designs and Outcomes Analyzed in Dry Needling Research in Sports

Among the various study designs, randomized experimental studies were the most prevalent, underscoring a commitment to rigorous scientific analysis within the field. Case reports also maintained a significant presence, likely contributing valuable anecdotal evidence and insights into individual cases. However, notably absent were cohort studies, which could offer deeper insights into the long-term effects and broader applicability of dry needling in athletic contexts. This deviation from common research practices in sports is intriguing, as observational studies typically dominate, whereas experimental studies are often less prevalent [51]. While experimental studies provide internal validity, nonexperimental studies, such as cohort studies, may offer information about longer exposure periods to dry needling [52], facilitating a better understanding of its efficacy and potential limitations as a long-term therapeutic practice in sports medicine. This, in turn, could guide more informed decision-making within the athletic community.

The reviewed studies on dry needling exhibited considerable variability in protocols, including differences in needle insertion duration (ranging from 5 to 15 min), number of insertions (from 3 to 12 needles), techniques used (e.g., “fast in, fast out” versus pistoning), and the incorporation of electrical stimulation or ultrasound guidance. These differences highlight a lack of standardization in dry needling dosages, with inadequate reporting of critical parameters such as insertion depth, total treatment time, and specific pain tolerance thresholds. To improve future research, it is recommended that protocols standardize these variables, clearly define dosage parameters, and incorporate consistent reporting guidelines to enhance reproducibility and clinical relevance.

Considering the outcomes of studies on dry needling among sports athletes, there appears to be a consistent focus (75%) on analyzing and reporting outcomes related to pain perception. This emphasis on pain assessment aligns with the primary objective of investigating the effects of dry needling. Specifically, dry needling targets trigger points, which are hyperirritable spots in skeletal muscle characterized by palpable nodules or taut bands of muscle fibers [53]. These trigger points can manifest as localized or referred pain, as well as muscle tension [54]. Needles are inserted into these trigger points to alleviate pain perception by disrupting the neural pathways involved in pain signaling [55]. Consequently, it is reasonable to anticipate that research outcomes regarding pain relief could be explored more comprehensively, regardless of whether they are conducted in postexercise or injury recovery contexts.

In studies focusing on athletic performance outcomes (58.3%), muscular strength and range of motion emerged as the most prevalent areas of investigation, whereas physiological adaptations were examined in fewer studies. It is hypothesized that impairments in athletic performance (e.g., postexercise), such as muscular performance, can be mitigated by managing pain reduction [56], while limitations in range of motion can be addressed by releasing tight muscles and promoting muscle relaxation [57]. Therefore, rather than testing the effects for improvements in athletic performance, studies examining physical performance have aimed to analyze how dry needling could alleviate impairments in physical performance postexercise or during injury processes.

#### Contexts of Studies on Dry Needling in Sports

In terms of the primary focus of research, the majority of postexercise studies employed experimental designs. Conversely, a more balanced distribution was observed in case reports regarding the reporting of pain-related outcomes and variations in athletic performance. Experimental studies involving injured athletes were relatively rare, likely owing to the complexities associated with recruiting injured athletes compared with the relatively simple process involved in case reports, which require fewer participants.

Considering the body regions targeted by interventions, particularly in connection with sports, a wide array of sports was represented (e.g., soccer, volleyball, running, handball, pole vaulting, and triathlons). Among these, the lower limbs were the most frequently addressed (58.3%), with a focus on muscles such as the gastrocnemius and thigh. Conversely, in the upper region, the shoulder muscles received the most attention (20.8%).

In sports modalities involving overhead movements (e.g., volleyball, handball, and tennis), the shoulder often experiences overuse, pain, and injuries [58], making it justifiable to prioritize research in this area. Meanwhile, hamstring injuries are commonly a focal point in sports requiring significant lower limb engagement (e.g., running and soccer) [59]. However, dry needling research in sports has predominantly concentrated on the gastrocnemius, possibly owing to the prevalence of studies involving runners who frequently experience muscle soreness in this area [59], as well as the thigh. It may be prudent to increase the focus on hamstring muscles in future studies to complement potential physiotherapy interventions or alleviate pain resulting from progressive increases in load, which often occur at specific points in the season (e.g., preseason).

### Dry Needling and Its Impact on Pain Alleviation in Sports Athletes

It is hypothesized that dry needling stimulates a cascade of neurophysiological events, including the release of endogenous opioids [60], such as enkephalins and endorphins [61], which act as natural pain relievers. Additionally, the insertion of needles into trigger points within the muscle tissue triggers a localized twitch response, which is believed to disrupt the dysfunctional motor endplate activity associated with chronic muscle pain [62]. Furthermore, dry needling has been shown to increase blood flow to the affected area [63], facilitating the delivery of oxygen and nutrients while aiding the removal of metabolic waste products. This enhanced circulation promotes tissue healing and reduces inflammation [64], possibly contributing to the attenuation of muscular pain symptoms.

It is justifiable to emphasize researching the pain symptoms experienced by athletes following interventions with dry needling. Some of the included studies demonstrated significant improvements in pain symptom alleviation, particularly in postexercise scenarios, as evidenced by studies such as those conducted by Benito-de-Pedro et al. [27], who reported reductions of 26.2% in pressure pain threshold, and Walsh et al. [20], who observed reductions of 4.3–10.7% in pressure pain threshold compared with control groups. However, studies such as those conducted by Cushman et al. [31], Dos Santos et al. [39], and Huguenin et al. [34] did not reveal significant effects when compared with control groups.

#### Studies Involving Athletes Experiencing Injuries or Musculoskeletal Pain or Dysfunction

In studies involving athletes experiencing injuries or musculoskeletal pain or dysfunction (e.g., shoulder pain, tennis elbow syndrome, scapular dyskinesia), significant positive effects in pain relief were observed compared with control groups. For instance, Ceballos-Laita et al. [38] reported that pain intensity decreased by 82.5%, Etminan et al. [45] found that the pain rate dropped by 88.4%, Kheradmandi et al. [46] observed that pressure pain threshold decreased by 0.7%, and Zarei et al. [7] showed a numerical pain rating score of − 75.9%. However, one study conducted by Janowski et al. [35] on ballet dancers with calf pain reported no significant differences between the treatment and control groups.

Additionally, in athletes experiencing pain and injuries, the presence of localized pain or injuries may provide more homogeneous targets for dry needling interventions. The focused nature of the injury site and the repetitive stressors inherent in athletic activities [60] may create a more conducive environment for consistent pain reduction outcomes with dry needling. Furthermore, the underlying pathology is often more clearly delineated in this population than in the population of nonathletes, allowing for a targeted and standardized approach to dry needling application, thus contributing to the observed effectiveness in reducing pain intensity across studies.

#### Studies Focusing on Postexercise Recovery

In postexercise scenarios, the efficacy of dry needling in pain management presents a landscape marked by inconsistent findings. The variability in outcomes can be attributed to multifactorial influences, notably the divergent physiological responses following exercise. Postexercise pain dynamics involve complex mechanisms, including inflammatory processes, neural sensitization, and tissue remodeling [65], which may interact with dry needling interventions in heterogeneous ways across individuals and study designs. Additionally, factors such as the timing of interventions relative to exercise cessation (which varied in terms of implementation time and subsequent follow-up periods ranging from 24 to 48 h or more) [30], the fitness level and intensity and duration of exercise [66], and individual pain perception thresholds [67] contribute to the observed discrepancies. Therefore, the efficacy of dry needling in postexercise recovery may depend on individual factors, highlighting the need for further research to identify those who may benefit most from the intervention [68].

In studies reporting significant improvements, the duration of dry needling interventions tended to be longer compared with postexercise studies, which often involved a single session. This trend is exemplified by, for instance, studies by Etminan et al. [45], which was composed of nine sessions over 3 weeks; Zarei et al. [7], which included 20 sessions over 4 weeks; and Kheradmandi et al. [46], which involved three sessions. Therefore, the duration and frequency of dry needling interventions could be concurrent factors that influence variations. Consequently, future research should explore and compare the frequency and duration of dry needling interventions to understand whether these factors can modulate how athletes manage pain or perceive the potential benefits of dry needling. Additionally, comparing possible extents is crucial, as the immediate acute effects can vary in duration depending on the athlete. This line of inquiry may unveil individualized thresholds or identify effective minimum doses for optimal outcomes.

### Dry Needling and Its Influence on Neuromuscular Function, Range of Motion, and Physiological Parameters

While the primary focus of dry needling in sports applications is pain management, their pain reduction could also enhance subsequent muscular performance or increase range of motion, thereby improving athletes’ physical capabilities during recovery. For instance, dry needling may facilitate improvements in muscle stiffness and motoneuron excitability of latent medial myofascial trigger points. However, the evidence is inconsistent and typically does not strongly support this hypothesis [56, 69, 70].

Studies tend not to show any significant effects of dry needling on muscle force production. For instance, Ceballos-Laita et al. [38] found no significant difference in internal and external rotation strength among handball players with shoulder pain compared with the control group. Additionally, Devereux et al. [33] revealed that, while maximal force and power were not significantly enhanced by dry needling compared with the control group, jump height improved 48 h after the intervention in male field sport athletes with latent myofascial trigger points in the rectus femoris and medial gastrocnemius muscles bilaterally. Furthermore, Etminan et al. [45] observed athletes with chronic tennis elbow and found no significant effects of dry needling compared with the regular physiotherapeutic approach in terms of grip strength. Similarly, Janowski et al. [35] observed no significant effects of dry needling on triceps surae torque among ballet dancers experiencing calf pain compared with the control group.

#### Studies Involving Athletes Experiencing Injuries or Musculoskeletal Pain or Dysfunction

In athletes with unilateral shoulder pain, Ceballos-Laita et al. [38] found that individuals exposed to dry needling exhibited more significant changes than the control group in internal rotation range of motion, external rotation, glenohumeral internal rotation deficit, external rotation gain, and extensibility. Conversely, Janowski et al. [35], who studied the effects on ballet dancers, found no statistical difference in lunge range of motion measurements between the dry needling and sham groups. Variations in study methodologies, such as the timing of effects analysis, which may have influenced the observed effects such as duration, along with differences in participant characteristics (e.g., shoulder pain and calf pain) and treatment durations, may have contributed to such discrepancies.

Concerning the balance of athletes dealing with pain and injuries, López-González et al. [36] found that basketball players with chronic ankle instability who underwent dry needling demonstrated statistically significant improvements in static postural control measures. These improvements were evidenced by decreased sway variability and center of pressure displacement among those who received dry needling compared with those who did not.

Additionally, Zarei et al. [7] reported that incorporating dry needling into regular physiotherapy interventions revealed clinically significant improvements in functional performance only in the dry needling group during dynamic balance testing. One possible explanation is that dry needling can enhance proprioception and sensory feedback necessary for maintaining balance [71]. However, regarding postexercise recovery, Benito-de-Pedro et al. [29] found no significant effects of dry needling on static plantar pressure compared with the ischemic compression group. Thus, further research is necessary to comprehend the mechanisms underlying the reported evidence. Comparative analyses are also needed to identify the influence of dry needling on the health of both injured and uninjured athletes.

Regarding physiological responses, Janowski et al. observed significant differences in mean temperature when investigating ballet dancers experiencing calf pain and comparing dry needling versus sham dry needling [35]. Specifically, both the right and left calves in the sham group, as well as the right calf in the dry needling group, exhibited noteworthy differences in temperature [35]. One potential explanation for the decrease in muscle temperature in dry needling is the activation of the descending pain inhibitory pathways, triggered by the insertion of the needle into the muscle tissue [72]. This activation can lead to the release of endogenous opioids and neurotransmitters such as serotonin and norepinephrine [60], which may induce local vasodilation and increase blood flow [63], consequently reducing muscle temperature.

#### Studies Focusing on Postexercise Recovery

Regarding postexercise recovery, a single session of dry needling was less effective than cold water immersion in restoring maximal force in Paralympic powerlifters according to Dos Santos et al. [39]. Conversely, Haser et al. [22] reported that soccer players exposed to dry needling experienced significant benefits in muscular endurance and hip flexion range of motion compared with the placebo group.

Considering range of motion, it has been hypothesized that dry needling can reduce muscle tone and tension by eliciting a local twitch response within trigger points, thus relaxing the affected musculature [73]. This reduction in muscle tension may allow for increased flexibility and range of motion in the targeted muscles [74]. In a postexercise context, Benito-de-Pedro et al. [29] revealed no significant differences in the dorsiflexion range of motion at the tibiofibular–talar joint or in static and dynamic plantar pressure alterations before and immediately after the intervention.

Regarding physiological responses, Aidar et al. [40] investigated dry needling’s impact on postexercise recovery in Paralympic powerlifters. Their findings revealed a decrease in systolic blood pressure immediately following dry needling treatment. However, a contrasting pattern emerged at 50- and 60-min post-recovery. The passive recovery method resulted in a significantly higher heart rate and doubled product values compared with both cold-water immersion and dry needling. Similarly, Dos Santos et al. [39] observed members of the same cohort and demonstrated that dry needling was the only intervention associated with increased interleukin (IL)-2 levels at various time points. Muscle thickness did not increase following dry needling; conversely, cold water application led to greater muscle thickness at 15 min and 2 h post-treatment. These results suggest that cold water immersion facilitates effective recovery up to 24 and 48 h postexercise, while dry needling appears to be a favorable option within the initial 24-h recovery window.

In a study focusing on postexercise recovery in triathletes, Benito-de-Pedro et al. [27] reported no statistically significant differences in thermography measurements between two treatment modalities: dry needling and ischemic compression. This lack of significant differences was consistent across the superficial zone adjacent to latent myofascial trigger points and corresponding anatomical locations with healthy soft tissue in the contralateral limb, before and after treatment.

### Adverse Effects of Dry Needling

Dry needling may pose potential risks to patients, albeit with minimal frequency and severity. For instance, in a survey involving 39 physiotherapists [15], commonly reported adverse effects included bruising, bleeding, pain during treatment, and post-treatment pain. While such mild adverse effects were frequently cited, significant adverse events were rare [15].

Among the 24 studies analyzed in our systematic review, only 3 explicitly documented adverse effects. Benito-de-Pedro et al.’s [29] study, which involved 17 athletes undergoing dry needling, noted two cases of local hematoma in the treatment area. Moreover, Brewster et al. [30] observed minimal and tolerable pain during needle insertion at various sites as the sole adverse reaction. Additionally, Huguenin et al. [34] noted that two subjects experienced syncopal responses to needling, but they recovered swiftly and completed the study. Meanwhile, one participant initially reported atypical chest pain, but this was resolved after a thorough medical evaluation, and the participant resumed the trial 2 days later.

Despite these isolated incidents, the majority of studies either reported no adverse effects or did not provide detailed documentation. However, there is inconsistency in reports of dry needling dosing parameters and adverse effects [75]. Without more comprehensive reporting, future studies will face significant limitations in replicating the methods of previous experiments and advancing the understanding of dry needling’s efficacy and safety profile.

### Study Limitations and Future Research

The current analysis of the identified methodological characteristics revealed a prevalent risk of bias stemming from the lack of blinding among participants and therapists, as well as insufficient detail regarding allocation concealment. These issues require significant consideration. Blinding, also known as masking, is a pivotal methodological tool that mitigates bias in research. The knowledge that participants or researchers have regarding the treatment administered can influence their behaviors and evaluations, potentially distorting the results. For example, participants who are aware that they are receiving an experimental treatment may report more favorable outcomes owing to placebo effects or subconscious biases. Likewise, if researchers are not blinded to the treatment, their expectations and interactions with participants could subtly sway the study’s outcomes.

Furthermore, allocation concealment is crucial in ensuring the impartial assignment of participants to various treatment groups. Inadequate concealment raises the risk of selection bias, by which researchers may consciously or inadvertently influence group assignments based on factors beyond randomization, thus compromising the study’s internal validity.

A review of the methodological report indicated that the significant limitations commonly found in the studies included in this systematic review pertain to the reporting of dosages, specific treatment procedures, and intervention details. This observation aligns with a previous systematic review [75] that underscored inconsistencies in reporting dry needling dosing parameters and adverse effects. The failure to accurately determine the effects of dry needling dosages on outcomes could introduce bias into decisions regarding its clinical efficacy or optimal dosage.

Future research should prioritize enhancing the quality of reporting on treatment procedures, dosages, and adverse effects, perhaps by adopting standardized reporting protocols tailored to these specific elements. Additionally, regarding the evidence gap map, it is crucial to expand the inclusion of elite and world-class athletes while also integrating more Paralympic athletes into studies. Furthermore, there is a need to incorporate a broader range of neuromuscular and physiological outcomes in experimental studies. Moreover, longitudinal cohorts are recommended to provide a comprehensive, long-term perspective on the effects of dry needling experienced by athletes.

## Conclusions

The current systematic review on dry needling in sports and sports recovery revealed that most studies are randomized experimental or case–control studies, while studies including cohorts for long-term analysis are conspicuously lacking. Among the primary populations included, those at the trained/developmental and national levels are the most studied, whereas there is a dearth of research on world-class athletes and specific populations such as Paralympians. The representation of different sexes is relatively balanced in dry needling studies.

Regarding methodological reporting, there is a clear inconsistency in reports of adverse effects and, notably, in the details of the procedures and dosages of dry needling interventions, which are crucial for ensuring replicability. Pain perception is the most prevalent outcome explored, while musculoskeletal functioning (including muscle strength, range of motion, and balance) is investigated relatively rarely and exhibits more diversity in reporting methods.

Most studies focused on testing the effects of dry needling in postexercise recovery or the treatment of pain or injuries. Overall, the evidence suggests that dry needling has significant positive effects on alleviating pain in athletes who are suffering from injuries or pain. However, the results regarding the benefits seen in healthy athletes in postexercise contexts are quite varied. Concerning the effects of dry needling on functioning, improved balance and range of motion are commonly observed benefits in injured athletes or those with pain. However, in healthy athletes, these effects are not significant. Dry needling appears to have limited effects on muscular force and physiological adaptations.

Therefore, there is a need for more studies of dry needling sports and increased diversity in such studies. Nevertheless, dry needling appears to be beneficial in reducing pain and improving certain functional parameters for athletes suffering from pain or injury. The effectiveness and a low level of adverse effects in these athletes were observed. Its efficacy appears to be less evident in healthy individuals than in individuals suffering from injuries or pain, but the results in this regard are ambiguous.

## Supplementary Information

Below is the link to the electronic supplementary material.

Supplementary file1 (DOCX 43 KB)

## Declarations

### Availability of data and material

All data are available by request to the corresponding author.
