# Supplementary material for: Dry Needling in Sports and Sport Recovery: A Systematic Review with an Evidence Gap Map
Source: Sports Med. 2025 Feb 10;55(4):811–44. doi: 10.1007/s40279-025-02175-9 (PMC12011942; doi:10.1007/s40279-025-02175-9)
Supplement: Supplementary file 1 — Supplementary file1 (DOCX 43 KB) [file 40279_2025_2175_MOESM1_ESM.docx]

Supplementary material 1. Full text screening.

| **AUTOMATIC SEARCHES** | **POPULATION** | **INTERVENTION** | **COMPARATOR** | **OUTCOMES** | **STUDY DESIGN** | **INCLUSION** |
| --- | --- | --- | --- | --- | --- | --- |
| Ingber, R. S. (2000). Shoulder impingement in tennis/racquetball players treated with subscapularis myofascial treatments. Archives of Physical Medicine and Rehabilitation, 81(5), 679-682. doi:10.1053/mr.2000.4418 | Y | Y | N | N | Y | N |
| Aidar, F. J., Dantas, E. F., Almeida-Neto, P. F., Neto, F. R., Garrido, N. D., Cabral, B. G., . . . Reis, V. M. (2022). Can Post-Exercise Hemodynamic Response Be Influenced by Different Recovery Methods in Paraplegic Sportsmen? Int J Environ Res Public Health, 19(3). doi:10.3390/ijerph19031772 | Y | Y | Y | Y | Y | Y |
| Benito-de-Pedro, M., Becerro-de-Bengoa-Vallejo, R., Losa-Iglesias, M. E., Rodríguez-Sanz, D., López-López, D., Cosín-Matamoros, J., ... & Calvo-Lobo, C. (2019). Effectiveness between dry needling and ischemic compression in the triceps surae latent myofascial trigger points of triathletes on pressure pain threshold and thermography: a single blinded randomized clinical trial. Journal of Clinical Medicine, 8(10), 1632. | Y | Y | Y | Y | Y | Y |
| Rainey, C. E. (2013). The use of trigger point dry needling and intramuscular electrical stimulation for a subject with chronic low back pain: a case report. Int J Sports Phys Ther, 8(2), 145-161. | N | Y | N | Y | Y | N |
| Dragoo, J. L., Wasterlain, A. S., Braun, H. J., & Nead, K. T. (2014). Platelet-rich plasma as a treatment for patellar tendinopathy: a double-blind, randomized controlled trial. Am J Sports Med, 42(3), 610-618. doi:10.1177/0363546513518416 | N | Y | Y | Y | Y | N |
| Jayaseelan, D. J., Moats, N., & Ricardo, C. R. (2014). Rehabilitation of proximal hamstring tendinopathy utilizing eccentric training, lumbopelvic stabilization, and trigger point dry needling: 2 case reports. J Orthop Sports Phys Ther, 44(3), 198-205. doi:10.2519/jospt.2014.4905 | N | Y | N | Y | Y | N |
| Benito-de-Pedro, M., Calvo-Lobo, C., López-López, D., Benito-de-Pedro, A. I., Romero-Morales, C., San-Antolín, M., ... & Rodríguez-Sanz, D. (2021). Electromyographic assessment of the efficacy of deep dry needling versus the ischemic compression technique in gastrocnemius of medium-distance triathletes. Sensors, 21(9), 2906. | Y | Y | Y | Y | Y | Y |
| Ortega-Cebrian, S., Luchini, N., & Whiteley, R. (2016). Dry needling: Effects on activation and passive mechanical properties of the quadriceps, pain and range during late stage rehabilitation of ACL reconstructed patients. Phys Ther Sport, 21, 57-62. doi:10.1016/j.ptsp.2016.02.001 | N | Y | N | Y | Y | N |
| Brewster, B. D., Valier, A. R. S., & Falsone, S. (2022). A Systematic Dry-Needling Treatment to Support Recovery Posttraining for Division i Ice Hockey Athletes: An Exploration Case Series. Journal of Athletic Training, 57(8), 788-794. doi:10.4085/1062-6050-0096.21 | Y | Y | N | Y | Y | Y |
| Stuhlman, C. R., Stowers, K., Stowers, L., & Smith, J. (2016). Current concepts and the role of surgery in the treatment of jumper's knee. Orthopedics, 39(6), e1028-e1035. doi:10.3928/01477447-20160714-06 | N | N | N | N | N | N |
| Tricás-Moreno, J. M., Lucha-López, M. O., Lucha-López, A. C., Salavera-Bordás, C., & Vidal-Peracho, C. (2016). Optimizing physical therapy for ankylosing spondylitis: a case study in a young football player. J Phys Ther Sci, 28(4), 1392-1397. doi:10.1589/jpts.28.1392 | N | Y | Y | N | Y | N |
| Gouttebarge, V., Veenstra, E., Goedegebuure, S., Frings-Dresen, M., & Kuijer, P. P. (2018). Professional football players at risk for non-acute groin injuries during the first half of the season: A prospective cohort study in the Netherlands. Journal of Back and Musculoskeletal Rehabilitation, 31(1), 15-21. doi:10.3233/BMR-150427 | Y | Y | Y | N | N | N |
| Knapik, D. M., Trem, A., Sheehan, J., Salata, M. J., & Voos, J. E. (2018). Conservative Management for Stable High Ankle Injuries in Professional Football Players. Sports Health, 10(1), 80-84. doi:10.1177/1941738117720639 | N | N | N | N | N | N |
| Shariat, A., Noormohammadpour, P., Memari, A. H., Ansari, N. N., Cleland, J. A., & Kordi, R. (2018). Acute effects of one session dry needling on a chronic golfer's elbow disability. J Exerc Rehabil, 14(1), 138-142. doi:10.12965/jer.1836008.004 | N | Y | N | Y | Y | N |
| Ceballos-Laita, L., Medrano-De-la-fuente, R., Estébanez-De-miguel, E., Moreno-Cerviño, J., Mingo-Gómez, M. T., Hernando-Garijo, I., & Jiménez-Del-barrio, S. (2021). Effects of dry needling in teres major muscle in elite handball athletes. A randomised controlled trial. Journal of Clinical Medicine, 10(18). doi:10.3390/jcm10184260 | Y | Y | Y | Y | Y | Y |
| Dembowski, S. C., Westrick, R. B., Zylstra, E., & Johnson, M. R. (2013). Treatment of hamstring strain in a collegiate pole-vaulter integrating dry needling with an eccentric training program: a resident's case report. Int J Sports Phys Ther, 8(3), 328-339. | Y | Y | N | Y | Y | Y |
| Devereux, F., OʼRourke, B., Byrne, P. J., Byrne, D., & Kinsella, S. (2019). Effects of Myofascial Trigger Point Release on Power and Force Production in the Lower Limb Kinetic Chain. J Strength Cond Res, 33(9), 2453-2463. doi:10.1519/jsc.0000000000002520 | Y | Y | N | Y | Y | Y |
| Dos Santos, W. Y. H., Aidar, F. J., de Matos, D. G., Van den Tillaar, R., Marçal, A. C., Lobo, L. F., . . . da Silva Júnior, W. M. (2021). Physiological and biochemical evaluation of different types of recovery in national level paralympic powerlifting. International Journal of Environmental Research and Public Health, 18(10). doi:10.3390/ijerph18105155 | Y | Y | Y | Y | Y | Y |
| Ansari, N. N., Alaei, P., Naghdi, S., Fakhari, Z., Komesh, S., & Dommerholt, J. (2020). Immediate Effects of Dry Needling as a Novel Strategy for Hamstring Flexibility: A Single-Blinded Clinical Pilot Study. J Sport Rehabil, 29(2), 156-161. doi:10.1123/jsr.2018-0013 | N | Y | N | Y | Y | N |
| Halle, R., Crowell, M., & Goss, D. (2020). DRY NEEDLING AND PHYSICAL THERAPY VERSUS PHYSICAL THERAPY ALONE FOLLOWING SHOULDER STABILIZATION REPAIR: A RANDOMIZED CLINICAL TRIAL. Int J Sports Phys Ther, 15(1), 81-102. | N | Y | Y | Y | Y | N |
| Escaloni, J., Mazloomdoost, D., & Young, I. (2023). Novel Orthobiologic Preparation and Regenerative Rehabilitation of a Complex Shoulder Injury in a Competitive Adolescent Female Athlete. Int J Sports Phys Ther, 18(1), 240-252. doi:10.26603/001c.68143 | Y | Y | N | Y | Y | Y |
| Etminan, Z., Razeghi, M., & Nezhad, F. G. (2019). The Effect of Dry Needling of Trigger Points in Forearm's Extensor Muscles on the Grip Force, Pain and Function of Athletes with Chronic Tennis Elbow. Journal of Rehabilitation Sciences and Research, 6(1), 27-33. doi:10.30476/jrsr.2019.44736 | Y | Y | Y | Y | Y | Y |
| Cushman, D. M., Cummings, K., Skinner, L., Holman, A., Haight, P., Brobeck, M., . . . Tang, C. (2021). Postrace Dry Needling Does Not Reduce Subsequent Soreness and Cramping-A Randomized Controlled Trial. Clinical Journal of Sport Medicine, 31(3), 225-231. doi:10.1097/JSM.0000000000000794 | Y | Y | N | Y | Y | Y |
| Haser C, Stöggl T, Kriner M, Mikoleit J, Wolfahrt B, Scherr J, Halle M, Pfab F. 2017. Effect of Dry Needling on Thigh Muscle Strength and Hip Flexion in Elite Soccer Players. Med Sci Sports Exerc. 2017 Feb;49(2):378-383. https://doi.org/10.1249/mss.0000000000001111 | Y | Y | Y | Y | Y | Y |
| Huguenin, L., Brukner, P., McCrory, P., Smith, P., Wajswelner, H., & Bennell, K. (2005). Effect of dry needling of gluteal muscles on straight leg raise: a randomised, placebo controlled, double blind trial. British journal of sports medicine, 39(2), 84. | Y | Y | Y | Y | Y | Y |
| Janowski JA, Phelan-Smith DML, Kroat Brady MN, et al. Acute effects of dry needling on myofascial trigger points in the triceps surae of ballet dancers: a pilot randomized controlled trial. Int. J. Sports Phys. Ther. 2021; 16:418–30 | Y | Y | Y | Y | Y | Y |
| Kamali, F., Sinaei, E., & Morovati, M. (2019). Comparison of Upper Trapezius and Infraspinatus Myofascial Trigger Point Therapy by Dry Needling in Overhead Athletes With Unilateral Shoulder Impingement Syndrome. J Sport Rehabil, 28(3), 243-249. doi:10.1123/jsr.2017-0207 | Y | Y | N | Y | Y | Y |
| Kheradmandi, A., Kamali, F., Ebrahimian, M., & Abbasi, L. (2021). Comparison between dry needling plus manual therapy with manual therapy alone on pain and function in overhead athletes with scapular dyskinesia: A randomized clinical trial. Journal of Bodywork and Movement Therapies, 26, 339-346. | Y | Y | Y | Y | Y | Y |
| Meshram, P., Vadhera, A. S., Sachdev, R., & McFarland, E. G. (2022). Delayed Recovery after Nonoperative Treatment of an Avulsion Fracture of the Ischial Tuberosity in an Adolescent Gymnast with a History of Growth Hormone Deficiency: A Case Report. Int J Sports Phys Ther, 17(5), 941-944. doi:10.26603/001c.37256 | Y | Y | N | N | Y | N |
| Sánchez-Infante, J., Bravo-Sánchez, A., Esteban-García, P., Jiménez, F., & Abián-Vicén, J. (2022). Changes in electromyographic activity of latent trigger points after a dry needling intervention: a randomised controlled trial. Physiotherapy, 117, 72-80. doi:10.1016/j.physio.2022.09.002 | N | Y | Y | Y | Y | N |
| Sharif, F., Ahmad, A., Gilani, S. A., Bacha, R., Hanif, A., & Arif, M. A. (2022). Efficacy of ultrasound guided dry needling as an adjunct to conventional physical therapy for patients with jumper's knee: A randomized controlled trial. Front Surg, 9, 1023902. doi:10.3389/fsurg.2022.1023902 | N | Y | N | Y | Y | N |
| Kim, D. C., Glenzer, S., Johnson, A., & Nimityongskul, P. (2018). Deep infection following dry needling in a young athlete: an underreported complication of an increasingly prevalent modality: a case report. JBJS Case Connector, 8(3), e73. | Y | Y | N | N | Y | N |
| Kaur, S., Maurya, U. K., & Shenoy, S. (2023). Recovery of two elite footballers from adductor strain with dry needling and eccentric strengthening: Two case studies. J Bodyw Mov Ther, 35, 332-336. doi:10.1016/j.jbmt.2023.04.043 | Y | Y | N | N | Y | N |
| Koehn, G., Jackson, L., Ablah, E., Okut, H., & Porter, A. (2023). Use of Ultrasound-Guided Tendon Fenestration and Injection Procedures for Treatment of Tendinosis. Kans J Med, 16, 258-260. doi:10.17161/kjm.vol16.18511 | N | Y | N | Y | Y | N |
| Sharif, F., Ahmad, A., & Gilani, S. A. (2023). Effectiveness of ultrasound guided dry needling in management of jumper's knee: a randomized controlled trial. Sci Rep, 13(1), 4736. doi:10.1038/s41598-023-31993-y | N | Y | Y | Y | Y | N |
|  |  |  |  |  |  |  |
| MANUAL SEARCHES |  |  |  |  |  |  |
| López-González, L., Falla, D., Lázaro-Navas, I., Lorenzo-Sánchez-aguilera, C., Rodríguez-Costa, I., Pecos-Martín, D., & Gallego-Izquierdo, T. (2021). Effects of dry needling on neuromuscular control of ankle stabilizer muscles and center of pressure displacement in basketball players with chronic ankle instability: A single-blinded randomized controlled trial. International Journal of Environmental Research and Public Health, 18(4), 1-14. doi:10.3390/ijerph18042092 | Y | Y | Y | Y | Y | Y |
| Mason, J. S., Tansey, K. A., & Westrick, R. B. (2014). Treatment of subacute posterior knee pain in an adolescent ballet dancer utilizing trigger point dry needling: a case report. Int J Sports Phys Ther, 9(1), 116-124. | Y | Y | N | Y | Y | Y |
| Osborne, N. J., & Gatt, I. T. (2010). Management of shoulder injuries using dry needling in elite volleyball players. Acupuncture in Medicine, 28(1), 42-45. doi:10.1136/aim.2009.001560 | Y | Y | N | Y | Y | Y |
| Bandy WD, Nelson R, Beamer L. Comparison of dry needling vs. sham on the performance of vertical jump. Int. J. Sports Phys. Ther. 2017; 12:747–51 | N | Y | Y | Y | Y | N |
| Patrick, R., McGinty, J., Lucado, A., & Collier, B. (2016). CHRONIC UCL INJURY: A MULTIMODAL APPROACH TO CORRECTING ALTERED MECHANICS AND IMPROVING HEALING IN A COLLEGE ATHLETE- A CASE REPORT. Int J Sports Phys Ther, 11(4), 614-626. | Y | Y | N | Y | Y | Y |
| Thompson, R., Prosell, M., & Timpka, T. (2021). Elite athletes' experiences of musculoskeletal pain management using neuroanatomical dry needling: A qualitative study in Swedish track and field. J Sci Med Sport, 24(1), 46-51. doi:10.1016/j.jsams.2020.07.004 | Y | Y | N | N | Y | N |
| Walsh, R., Kinsella, S., & McEvoy, J. (2019). The effects of dry needling and radial extracorporeal shockwave therapy on latent trigger point sensitivity in the quadriceps: A randomised control pilot study. J Bodyw Mov Ther, 23(1), 82-88. doi:10.1016/j.jbmt.2018.02.010 | Y | Y | Y | Y | Y | Y |
| Westrick, R. B., Zylstra, E., Issa, T., Miller, J. M., & Gerber, J. P. (2012). Evaluation and treatment of musculoskeletal chest wall pain in a military athlete. International Journal of Sports Physical Therapy, 7(3), 323. | Y | Y | N | Y | Y | Y |
| Zarei, H., Bervis, S., Piroozi, S., & Motealleh, A. (2020). Added Value of Gluteus Medius and Quadratus Lumborum Dry Needling in Improving Knee Pain and Function in Female Athletes With Patellofemoral Pain Syndrome: A Randomized Clinical Trial. Arch Phys Med Rehabil, 101(2), 265-274. doi:10.1016/j.apmr.2019.07.009 | Y | Y | Y | Y | Y | Y |
| Benito-de-Pedro, M., Becerro-de-Bengoa-Vallejo, R., Elena Losa-Iglesias, M., Rodríguez-Sanz, D., López-López, D., Palomo-López, P., ... & Calvo-Lobo, A. C. (2020). Effectiveness of deep dry needling vs ischemic compression in the latent myofascial trigger points of the shortened triceps surae from triathletes on ankle dorsiflexion, dynamic, and static plantar pressure distribution: A clinical trial. Pain Medicine, 21(2), e172-e181. | Y | Y | Y | Y | Y | Y |
